# Introduction and persistence of tularemia in Bulgaria

**DOI:** 10.1080/20008686.2017.1323532

**Published:** 2017-06-13

**Authors:** Mohammad Khan

**Affiliations:** ^a^ Department of Microbiology, School of Medicine, University of Dammam, Dammam, Saudi Arabia

Myrtannas et al. [1] studied new outbreaks of tularemia in Bulgaria. According to the evidence presented and the data corroborated with by the authors, the new outbreaks occurred after a lull of over three decades in a wide area (1000–1500 km^2^) abutting the Balkan Mountain Range (BMR) near the Bulgarian capital, Sofia. The new epicenter of the tularemia outbreak was 300 km west of the epicenter of the previous outbreak in Eastern Bulgaria. The authors undertook to track the source of the outbreak using molecular methods (e.g. analyses of single-nucleotide polymorphisms of the outbreak isolate).Their study is remarkable for, among other things, the relative lack of the application of traditional epidemiological methods in tandem with the molecular methods. They made no definite conclusion.

The authors of the study referred to above did not mention the total number of cases detected during in new outbreaks, making one wonder how the authors defined an outbreak.[2] Also, it was not clear what clinical form of tularemia was detected during the outbreak. This is important, considering that the authors [1] postulated that the high BMR was an inviolable physical barrier to the transmission of *Francisella tularensis* (FT) from the old to the new foci of outbreaks. However, the high BMR may not necessarily be a physical barrier to the aerosolized transmission of FT.[3]

The authors [1] suspected muskrats as the most likely sources of the outbreak strains of FT, yet provided no epidemiologic evidence suggestive of changing activities [4,5] of the animal before (e.g. distinctive muskrat tracks inland or dome-shaped lodges of the animal on water-logged surfaces) or after the outbreaks (e.g. increased number of dead muskrats). Also, absent is any information on the seasonality of the outbreaks studied by Myretennas et al. [1]. Seasonality of the outbreak would have given important clues about the sources of the outbreak strains of FT.[5]

The study presented by Myrtannas et al. [1] shows that the genomic study of the outbreak strains of FT uncoupled with traditional epidemiologic approach may not enough to track the source of the pathogen.
